# Evaluating the probiotic effects of spraying *lactiplantibacillus plantarum* P-8 in neonatal piglets

**DOI:** 10.1186/s12866-024-03332-2

**Published:** 2024-07-09

**Authors:** Guoqiang Yao, Zhixin Zhao, Chengcong Yang, Bin Zuo, Zhihong Sun, Junjun Wang, Heping Zhang

**Affiliations:** 1 Key Laboratory of Dairy Biotechnology and Engineering, Ministry of Education, Key Laboratory of Dairy Products Processing, Ministry of Agriculture and Rural Affairs, Inner Mongolia Key Laboratory of Dairy Biotechnology and Engineering, Inner Mongolia Agricultural University, Huhhot, Inner Mongolia China; 2 State Key Laboratory of Animal Nutrition, College of Animal Science and Technology, China Agricultural University, Beijing, China

**Keywords:** *Lactiplantibacillus plantarum* P-8, Neonatal piglets, Growth performance, Gut microflora, Metagenome

## Abstract

**Background:**

Gut microbes play an important role in the growth and health of neonatal piglets. Probiotics can promote the healthy growth of neonatal piglets by regulating their gut microbes. The study investigated the effects of spraying *Lactiplantibacillus plantarum* P-8 (*L. plantarum* P-8) fermentation broth on the growth performance and gut microbes of neonatal piglets.

**Results:**

The animals were randomly divided into probiotics groups (109 neonatal piglets) and control groups (113 neonatal piglets). The probiotics group was sprayed with *L. plantarum* P-8 fermented liquid from 3 day before the expected date of the sow to the 7-day-old of piglets, while the control group was sprayed with equal dose of PBS. Average daily gain (ADG), immune and antioxidant status and metagenome sequencing were used to assess the changes in growth performance and gut microbiota of neonatal piglets. The results showed that *L. plantarum* P-8 treatment significantly improved the average daily gain (*P* < 0.05) of neonatal piglets. *L. plantarum* P-8 increased the activities of CAT and SOD but reduced the levels of IL-2 and IL-6, effectively regulating the antioxidant capacity and immunity in neonatal piglets. *L. plantarum* P-8 adjusted the overall structure of gut microflora improving gut homeostasis to a certain extent, and significantly increased the relative abundance of gut beneficial bacteria such as *L. mucosae* and *L. plantarum*.

**Conclusion:**

Spraying *L. plantarum* P-8 can be a feasible and effective probiotic intervention not only improving the growth of neonatal piglets, regulating the antioxidant capacity and immunity of neonatal piglets, but also improving the gut homeostasis to a certain extent.

**Supplementary Information:**

The online version contains supplementary material available at 10.1186/s12866-024-03332-2.

## Introduction

In recent years, with the emergence of metagenomics and high-throughput sequencing, the gut microbiota has gained much attention [1]. The gut is the largest ecosystem in mammals [2], involving gut microbiota-mediated metabolic regulatory activities that significantly affect the host’s health [3]. Gut microbes play a vital role in pig health. Early colonization of the gut microbiota can majorly influence the establishment of gut microbiota, significantly affecting the growth, development, and health of neonatal piglets [4]. The gut microbiota has a long-term co-evolution with the host influenced by external environmental factors and lifestyle [5]. During the long-term evolution, the harmonious relationship between the gut microbiota and host maintained the microecological balance under the influence of environmental factors. The environment is one of the key factors [6], especially in early life, that significantly influences the structure of gut microbiota [7]. The living environment of neonatal piglets, especially during the early colonization of gut microbiota, is an important factor affecting the health of their gut microbiota. For instance, neonatal piglets are born from the safe and stable uterine environment in the sow that later changes to a complex external environment [8]. Meanwhile, with the continuous changes in the environment, the gut microbiota of the neonatal piglets also changes successively with the growth of the piglets, finally reaching a mature and stable state that can better adapt to the environment [9].

In China, pork is an indispensable part of people’s diet, and its demand has a crucial impact on the national economy and people’s livelihood. The neonatal period, an important stage in the growth and development of pigs, has a crucial impact on the survival rate of commercial pigs affecting the pork supply. The neonatal period is the main stage of early microbial colonization in the gut involving the interactions between the growth environment and microbiota in breast milk, which shape the early gut microbiota structure of neonatal piglets. In the neonatal stage, the gut function and immune system of neonatal piglets are not yet perfect and the gut can be easily invaded by pathogenic bacteria causing high animal mortality [10]. The probiotic intervention has broad application prospects to promote the normal growth of neonatal piglets by establishing their gut microbiota. Probiotics are a class of microorganisms that can reach the gut alive after ingestion in sufficient amounts, producing beneficial effects on the host [11]. Studies have shown that supplementing sows with probiotics during late gestation and lactation can effectively increase the average daily gain of piglets and improve intestinal epithelial morphology and intestinal immunity [12]. Probiotic intervention before and after the weaning of piglets effectively prevents gut infections [13]. Meanwhile, probiotic intervention adjusts the balance of gut microbiota promoting the absorption of nutrients and thereby growth in neonatal piglets [14].

*L. plantarum* P-8 was isolated in 2005 from the natural fermentation of sour cow’s milk in the grassland of Urad Middle Banner, Bayannur City [15]. A large number of animal experiments have shown that *L. plantarum* P-8 supplementation can improve the immune response and antioxidant capacity of the body, and has the effect of inhibiting intestinal pathogens [16–18]. What’s more, dietary supplementation with *L. plantarum* P-8 may ameliorate stress and improve antioxidant capacity and growth performance in weaned piglets, accompanied by beneficial changes to the gut microbiota [19].

Previously, traditional methods, such as gavage, were used for probiotic interventions, which often led to different degrees of stress-induced dysregulation causing injury, disease, and death in neonatal piglets. Therefore, this study used spraying of *L. Plantarum* P-8 fermentation broth in the farrowing room to assess its effect on gut microbiota and growth characteristics of neonatal piglets. The spraying method not only solves the stress problem from traditional gavage method, but also improves the application of probiotics in pig breeding industry.

## Materials and methods

### Ethics and consent to participate

All animal procedures were approved by the Animal Ethics Committee of China Agricultural University, and obtained informed consent from the owners to use the animals in this study. All animal experimental protocols were performed in accordance with the Guidelines for Care and Use of Laboratory Animals of China Agricultural University, and approved by the Relevant standards of animal welfare at China Agricultural University (Beijing, China).

### Animals and production of *L. plantarum* P-8 fermentation broth

The experimental animals were neonatal piglets born from cross between Landrace × Large White binary sows and Duroc boars. A total of 20 pregnant sows with similar parity and body condition were selected and randomly divided into 10 each in the probiotics group (LP) and the control group (CON). Pregnant sows in the probiotic group gave birth to 109 neonatal piglets, of which 95 neonatal piglets were used for body weight measurement, 6 neonatal piglets were slaughtered for sampling, 2 neonatal piglets were died, and the remaining 6 neonatal piglets were disease piglets. Pregnant sows in the control group gave birth to 113 neonatal piglets, of which 98 neonatal piglets were used for body weight measurement, 6 neonatal piglets were slaughtered for sampling, 4 neonatal piglets died, and the remaining 5 neonatal piglets were disease piglets (Table S1).

This study used the *L. plantarum* P-8 strain was provided by the Key Laboratory of Dairy Biotechnology and Engineering, Ministry of Inner Mongolia Agricultural University (Hohhot China). And *L. plantarum* P-8 has been publicly preserved in China General Microbiological Culture Collection Center (CGMCC, the number of CGMCC No.5486). The cryopreserved *L. plantarum* P-8 was inoculated at 10% inoculum in conical flasks containing MRS liquid medium (pH 5.6–6.2) for passaging activation for 24 h to obtain the strain fermentation broth. The strain fermentation broth was inoculated into conical flasks containing MRS liquid medium (pH 5.6–6.2) at 10% inoculum, and the number of viable strains was expanded at 33–37 °C for 13–15 h to make the number of viable strains in the final *L. plantarum* P-8 fermentation broth reach 1 × 10^10^ CFU/mL. Two hundred and fifty milliliter of *L. plantarum* P-8 fermentation broth was prepared every day to meet the amount used on the day of the experiment. In order to ensure the activity of strains in the *L. plantarum* P-8 fermentation broth, the *L. plantarum* P-8 fermentation broth must strictly abide by the principle of current preparation and current use.

### Experimental design, management and sample collection

The experiment lasted for 24 days (3 days before the sow’s expected date of birth to 21 days after the piglets were born). In the experiment, the probiotic intervention of neonatal piglets was carried out by spraying *L. plantarum* P-8 fermentation broth [20, 21]. The *L. plantarum* P-8 fermentation broth spraying period started from 3 days before the sow’s expected date of birth to 7 days after the piglets were born. Ma showed that complex probiotics (*L. plantarum* B90 ≥ 1.0 × 10^8^ CFU/g and *Saccharomyces cerevisiae* P11 ≥ 0.2 × 10^8^ CFU/g) were supplemented in the basic diet of pregnant sow significantly improved immune and antioxidant function in suckling piglets via modifying gut microbiota [22]. Yu showed that supplementation of piglets with *L. plantarum* P-8 (1 × 10^8^ CFU/g) improved growth performance of weaned piglets [19]. Therefore, in combination with the current research status and the total number of neonatal piglets in our experiment, the *L. plantarum* P-8 fermentation broth spraying dosage every time was finally determined to be 100 ml (1 × 10^10^ CFU/ml) in order to ensure the reliability of the experimental results. The probiotics group was sprayed with 100 ml of *L. plantarum* P-8 fermentation broth each time at 9:00 and 21:00 every day during the spraying period, and the fermentation broth was diluted with sterile PBS (*L. plantarum* P-8 fermentation broth: PBS = 1:2) before spraying to reduce the viscosity and facilitate spraying. The control group was sprayed with equal dose of PBS. The spraying area included the udder of the sow and the range activities of piglets (farrowing bed and fence).

These experiments were carried out at the Animal Test Base of the Feed Industry Center of the Ministry of Agriculture (Chengde China). The room temperature in the delivery room is kept at 24 ∼ 27℃, and the humidity is 50% ∼ 70%. Each delivery bed is equipped with heat-preservation lamps to keep the temperature of the piglet’s activity range at about 30℃. Lactating sows are reared in 2.2 × 1.8 m^2^ maternity bed with semi-leaky plastic floor, equipped with stainless steel adjustable trough and nipple drinking fountain for sows. The health monitoring of the experimental animals was carried out before the experiment to ensure that the experimental animals were in a healthy state. During the whole test period, pregnant sows fed a corn-soybean meal basal diet without any antibiotics or probiotics, and the piglets were not treated with tooth clipping or tail docking, and the male piglets were not castrated. Piglets were allowed *ad libitum* suckling, drinking water, and feeding at 10-day-old and were weaned at 21-day-old. The immunization program and other feeding management were consistent with the pig farms. The birthing beds were cleaned every day and the appetite, spirit, and feces of the piglets were observed.

Six piglets closest to the median weight for each replicate were randomly selected from each group to collect fecal samples using sterile 20 mL centrifuge tubes at 3-day-old and 7-day-old. On 8-day-old, animal blood anticoagulant tube (Shanghai Yuyan Scientific Instrument Co., LTD, China) was used to collect the anterior vena cava blood of the above 6 piglets. The collected venous blood samples were centrifuged at 3500 r/min for 15 min by a High-speed refrigerated centrifuge (Eppendorf, Germany) to obtain the respective plasma samples. Then 6 piglets whose venous blood samples have been collected were slaughtered. Immediately after the slaughtered, the ileal mucosa samples and liver tissues samples were collected using sterile 50 mL centrifuge tubes. All samples were transferred to liquid nitrogen after collection, and then transported back to the laboratory for storage at -80℃ until analysis.

### Determination of growth performance of neonatal piglets

In total, 193 neonatal piglets (95 and 98 in the probiotics and control groups, respectively) were used for the comparison of average daily gain, excluding those that died, slaughtered, developed a disease, or showed abnormal growth. There are three stages of growth in piglets from 0-21d. The first stage denotes from 0-7d; the second stage denotes from 7-14d, and the third state denotes from 15-21d. The death rate (Formula 1) of each group was calculated during the study period, and the average daily gain (Formula 2) were measured on 7-day-old, 14-day-old and 21-day-old.

#### Formula 1

Death rate (%) = (total number of piglets that died in the group / total number of piglets in the group) × 100%.

#### Formula 2

Average daily gain (g/d) = (average body weight at the end of each period - average body weight at the beginning of each period) / total number of days in each period.

### Detection of immune and antioxidant markers in neonatal piglets

The plasma samples were tested for total antioxidant capacity (T-AOC, U/mg); catalase (CAT, U/mg); superoxide dismutase (SOD, U/mg); glutathione peroxidase (GSH-Px, U/mg); glutathione (GSH, µmol/g); malondialdehyde (MDA, nmol/mg); immunoglobulin A (IgA, g/g); immunoglobulin G (IgG, g/g); immunoglobulin M (IgM, g/g); interferon-γ(IFN-γ, pg/mg); tumor necrosis factor-α (TNF-α, pg/mg); interleukin-1β(IL-1β, pg/mg); interleukin-6 (IL-6, pg/mg); interleukin-2 (IL-2, pg/mg); interleukin-4 (IL-4, pg/mg) and interleukin-10 (IL-10, pg/mg) using respective detection kits provided by Nanjing Jiancheng Bioengineering Research Institute (Nanjing China) following the manufacturer’s instructions. Likewise, ileal mucosa samples were also tested for T-AOC, CAT, SOD, GSH-Px, GSH, MDA, IgA, IgG, IgM, IFN-γ, TNF-α, IL-1β, IL-6, IL -2, IL-4, and IL-10. The liver samples were tested for T-AOC, CAT, SOD, GSH-Px, GSH, and MDA.

### DNA extraction and shotgun metagenomic sequencing

DNA was extracted from fecal samples for metagenomics using the QIAGEN QIAamp Fast DNA Stool Mini Kit (Qiagen GmbH, Hilden, Germany) following the manufacturer’s instructions. The isolated DNA was checked for purity and concentration by the Nanodrop spectrophotometer, and Qubit® dsDNA Assay Kit in combination with a Qubit® 2.0 fluorometer (Life Technologies, CA, USA). The integrity of the DNA was checked by 1.0% agarose gel electrophoresis. Only the DNA samples > 20 ng/µL and an optical density ratio (260 to 280 nm) of 1.8-2.0 were processed for paired-end sequencing on the Illumina Hiseq 4000 sequencing platform (Tianjin Novogene Technology Co., Ltd., Tianjin, China).

### Bioinformatics of metagenomic data

In total, 24 fecal samples were sequenced, generating 0.371 Tbp of high-quality paired-end reads (15.47 ± 0.75 Gbp raw metagenomic reads per sample). Quality control of raw metagenomic reads was conducted by the KneadData quality control pipeline (http://huttenhower.sph.harvard.edu/kneaddata; v0.7.5) to filter out low-quality reads (length < 60 nt) by Trimmomatic (a flexible trimmer for sequence data generated by Illumina [23]). The high-quality reads were then aligned to the porcine DNA sequence using Bowtie2 (v2.3.5.1) [24], and the reads showing high similarity were removed. After the quality control filtering, a total of 0.368 Tbp of clean data (15.32 ± 0.74 Gbp clean metagenomic reads per sample, Table S2-S3) were obtained for downstream analysis.

The quality-controlled metagenomic data were analyzed using HUMAnN2 [25]. Program MetaPhAn2 was used to annotate the composition of microorganisms in respective samples. The pan-genome nucleotide sequences in each sample were aligned with the ChocoPhIAn database by Bowtie 2 software. The translated protein sequences were aligned with the UniRef 50 database using Diamond software. Finally, based on the MetaCyc database, the HUMAnN core algorithm was used to compare the aligned genome and protein sequences for cross-comparison analysis to obtain gene family, pathway abundance, and pathway coverage files for subsequent analysis.

### Statistical analysis

The change in richness and alpha diversity of microbial species communities and metabolic pathways were calculated using the Vegan package in R software (v.4.0.2). Wilcoxon tests were used to make a comparison of significant differences between groups. Principal coordinates analysis (PCoA) was performed to evaluate overall changes in the gut microbiota community structure and metabolic pathways, and analysis of similarities (PERMANOVA, 999 permutations) was performed to evaluate differences between the groups. R software combined with LDA Effect Size (LEfSe) analysis was used to screen out dominant and differential bacteria. Correlation analysis was used to explore functional correlations with microorganisms. The random forest model (RFM) was used to screen out the high-contributing species. All graphical presentations were generated under the R and Adobe Illustrator environment.

## Results

### *L. plantarum* P-8 reduced mortality and significantly promoted the weight of neonatal piglets

Mortality, a complex phenomenon that is affected by many factors, is an important indicator of neonatal piglets’ adaptability to the growing environment. The influence of *L. plantarum* P-8 on the viability of neonatal piglets was evaluated by calculating the mortality of neonatal piglets. The mortality in the probiotic group was lower (1.83%; 2/109) than that in the control group (3.54%; 4/113), and the chi-squared test found no significant difference in mortality between the two groups (Table 1).

**Table 1:** Mortality data of neonatal piglets

Project	Probiotic group (LP)	Control group (CON)
Total piglets	109	113
Total deaths	2	4
Mortality	1.83%	3.54%
Chi-Squared Test: x^2^ = 0.136 *P* = 0.712		

Comparison of mortality rates between the LP and CON

**Fig. 1:**
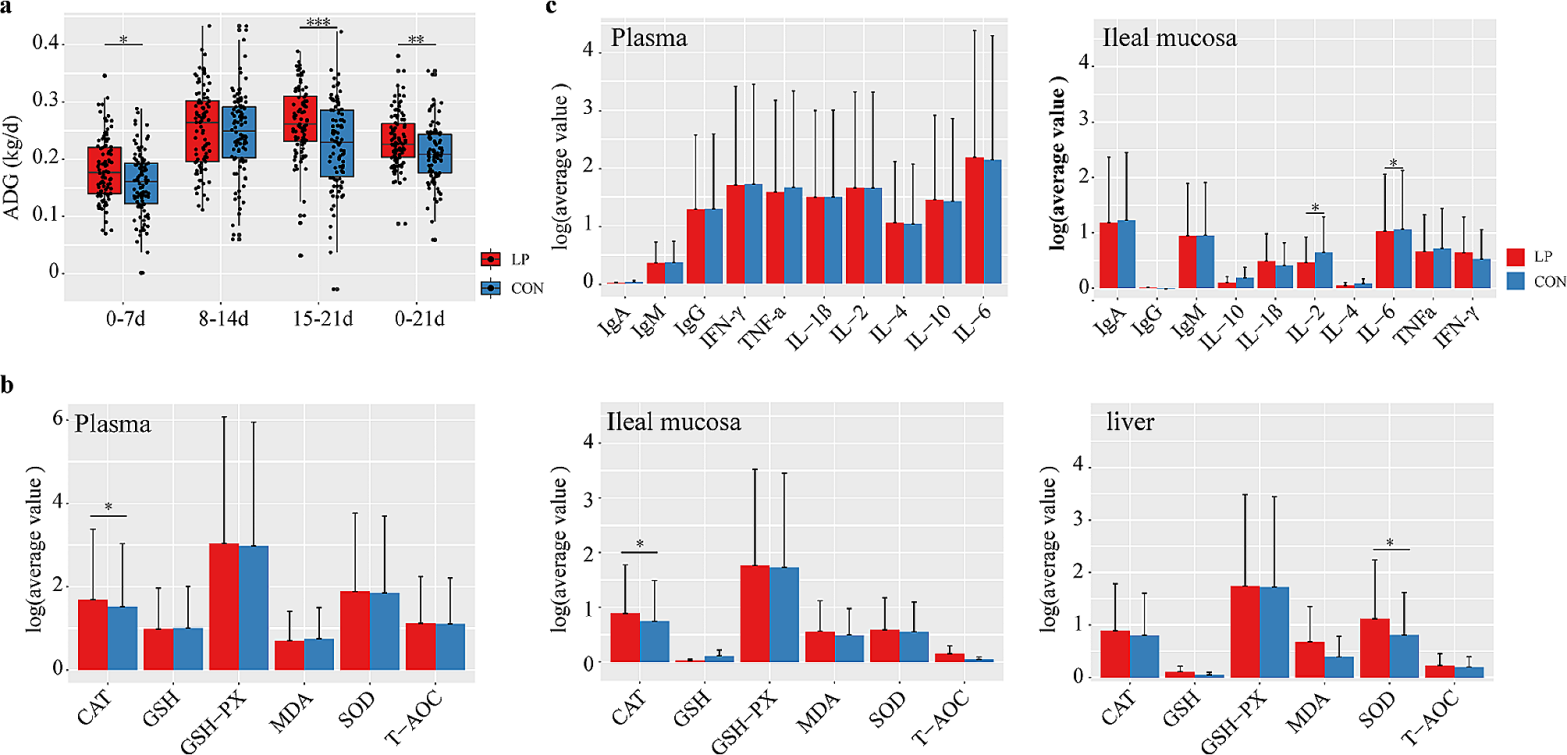
(**a**) Comparison of average daily weight gain between the two groups at the respective stage. Comparison of the changes in (**b**) antioxidant factors in plasma, ileal mucosa, and liver and (**c**) immune status between the two groups in 8-day-old piglets. Red and blue represent the probiotic group (LP) and control group (CON), respectively

To further examine the *L. plantarum* P-8 effect on the growth of neonatal piglets, the change in average daily gain (ADG) was estimated (Fig. 1a, Table S4). Excluding the slaughtered, dead and disease piglets, 95 and 98 piglets in the probiotic and control groups were compared for the change in the ADG, respectively. The ADG was significantly higher in the probiotic group than in the control group during 0-7d (*P* < 0.05), and the same trend was observed during 15-21d (*P* < 0.05). However, there was no significant difference of ADG between probiotic group and control group during 8-14d. It was worth noting that the ADG was significantly higher in the probiotic group than in the control group during 0-21d (*P* < 0.05). Overall, *L. plantarum* P-8 significantly promoted the ADG of neonatal piglets.

### *L. plantarum* P-8 effectively regulate antioxidant capacity and immunity in neonatal piglets

To evaluate the probiotic effect of *L. plantarum* P-8 spraying on the antioxidant capacity and immunity of neonatal piglets, the change in levels of antioxidant indexes and immune factors were estimated (Fig. 1b-c, Table S5-S7). The results showed that among the measured activities, the levels of CAT in plasma and ileal mucosa and SOD in the liver were significantly higher in the probiotic group compared with the control group (*P* < 0.05). Among the immune factors, IL-2 and IL-6 in the ileal mucosa were significantly lower in the probiotic group compared with the control group (*P* < 0.05). All the other tested indicators did not show a significant difference. These results suggested that *L. plantarum* P-8 may could regulate antioxidant capacity and immunity in neonatal piglets.

### *L. plantarum* P-8 significantly changed gut microbiota structure and increased beneficial bacteria in neonatal piglets

**Fig. 2:**
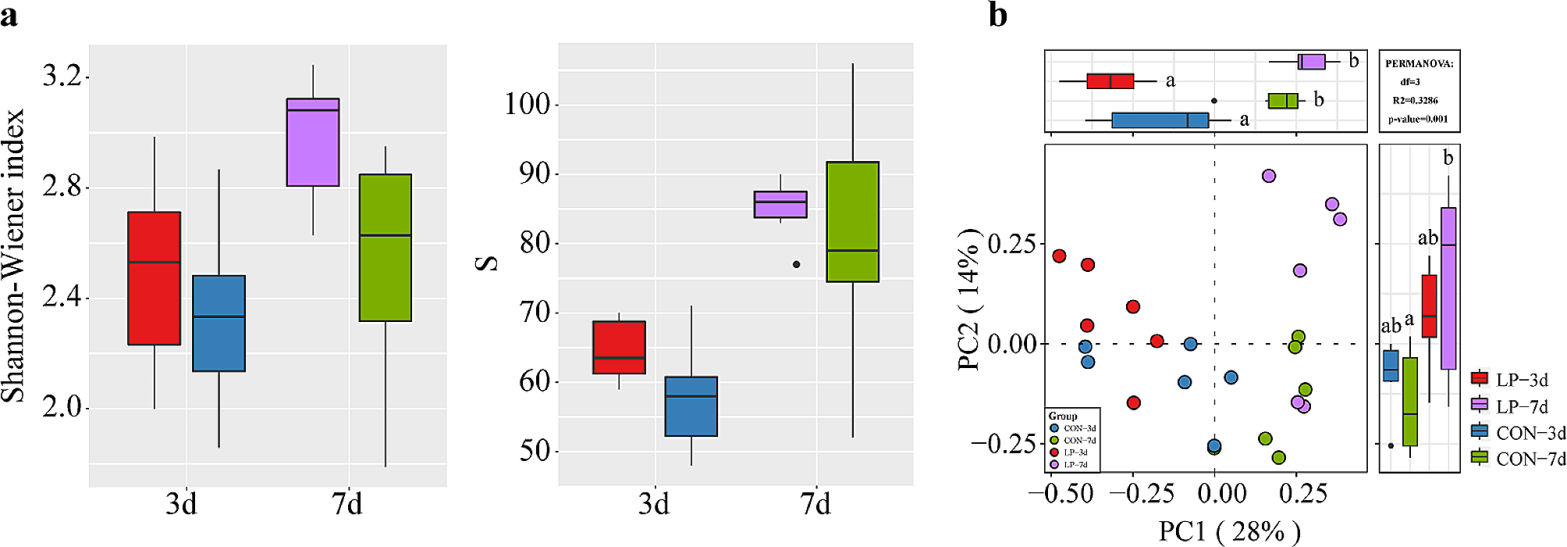
Diversity analysis of fecal samples in neonatal piglets. (**a**) Comparative box diagram showing the Shannon and Simpson indices indicating the bacterial diversity and richness of fecal samples. (**b**) Principal co-ordinates analysis (PCoA) was performed based on bray distance. PCoA score plots are based on principal components 1 and 2 (at the species level). Red (LP-3d) and blue (CON-3d) represent the probiotic and control groups of 3 d old piglets, respectively. Purple (LP-7d) and green (CON-7d) represent the probiotic and control groups of 7-day-old piglets, respectively

Alpha diversity analysis of the gut microbiota in neonatal piglets at the species level (Fig. 2a). Seven-day-old piglets showed higher diversity and richness of gut microbiota than the 3-day-old piglets, which indicated the gradual establishment of gut microbiota. Notably, there was no significant difference in the diversity and richness of gut microbiota between the probiotic and control groups. Beta diversity (shown by PCoA and PERMANOVA) showed a significant difference on gut microbiota structure in the 7-day-old piglets (Fig. 2b). This indicated that during the establishment of gut microbiota, the two groups showed a clear trend of separation. These results suggested that *L. plantarum* P-8 broth did not significantly change the diversity but changed the structure of gut microbiota in neonatal piglets.

**Fig. 3:**
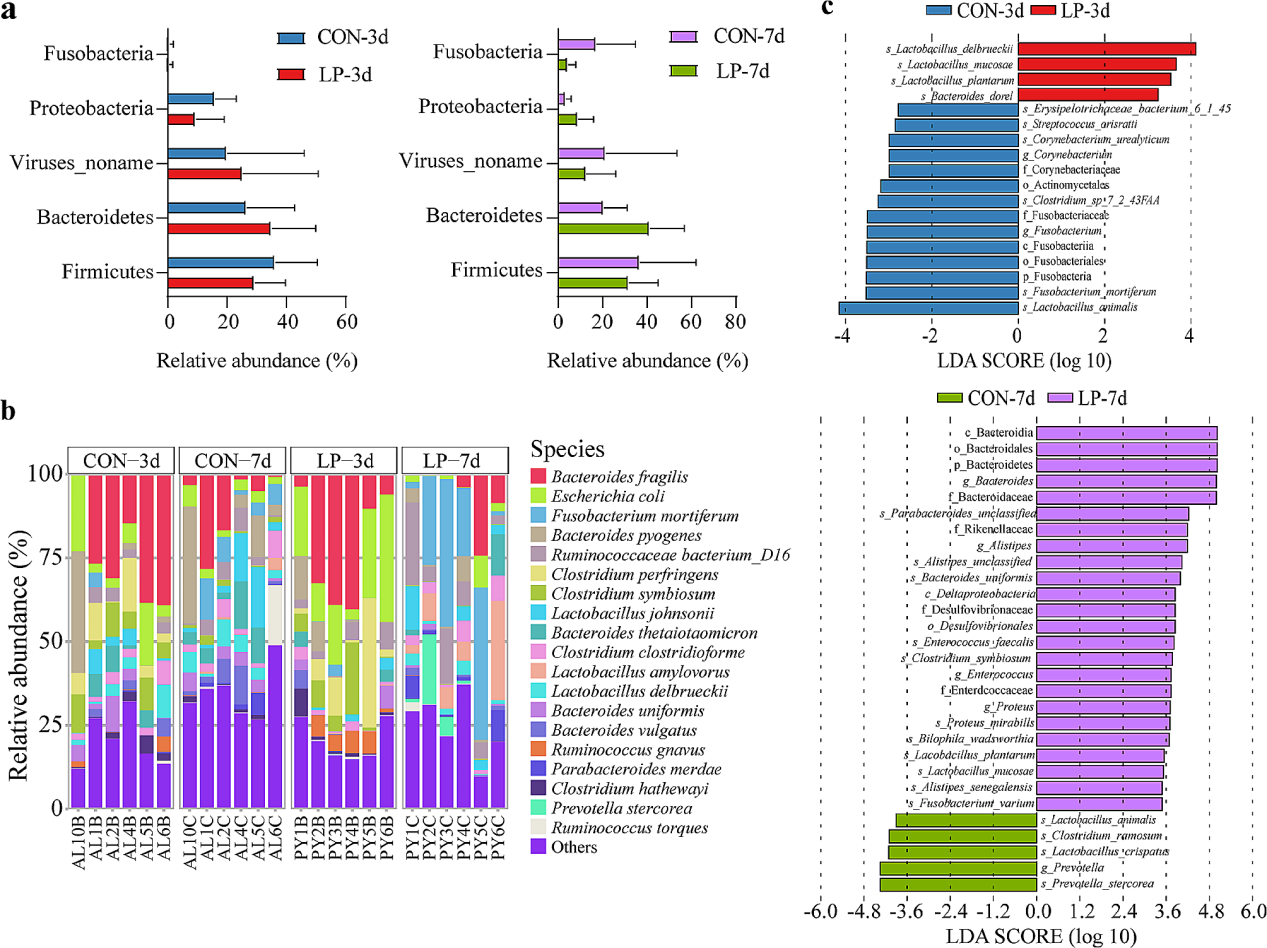
Screening of dominant and differential bacteria. (**a**) Change in microbiota community structure in fecal samples at the phylum level in 3 and 7-day-old piglets between the two groups. Only phyla with > 1% abundance are shown. (**b**) Change in microbiota community structure at the species level. Only species with > 1% abundance are shown. (**c**) Linear discriminant analysis (LDA) of the effect size for taxa with differential abundance in the two groups at 3-day-old and 7-day-old piglets. Red (LP-3d) and blue (CON-3d) represent the probiotic and control groups of 3-day-old piglets, respectively. Purple (LP-7d) and green (CON-7d) represent the probiotic and control groups of 7-day-old piglets, respectively

At the phylum level, Firmicutes, Bacteroidetes, Proteobacteria, and Fusobacteria were the dominant phyla with a relative abundance of > 1% (Fig. 3a). Nineteen dominant bacterial species with a relative abundance of > 1%, were identified among a total of 201 species, including the most dominant *Bacteroides fragilis* (12.89%), *Escherichia coli* (6.51%), *Fusobacterium mortiferum* (5.64%) and *Bacteroides pyogenes* (4.69%) (Fig. 3b). Importantly, the differential analysis by LEfSe revealed that the relative abundance of *Lactobacillus delbrueckii*, *Lactobacillus mucosae*, and *Lactobacillus plantarum* were significantly higher in the probiotic group than in the control group for the 3-day-old piglets. For the 7-day-old piglets, the relative abundance of Bacteroides, Alistipes, *L. mucosae and L. plantarum* were significantly higher in the probiotics group than in the control group (Fig. 3c).

### *L. plantarum* P-8 significantly altered metabolic pathways in neonatal piglets

**Fig. 4:**
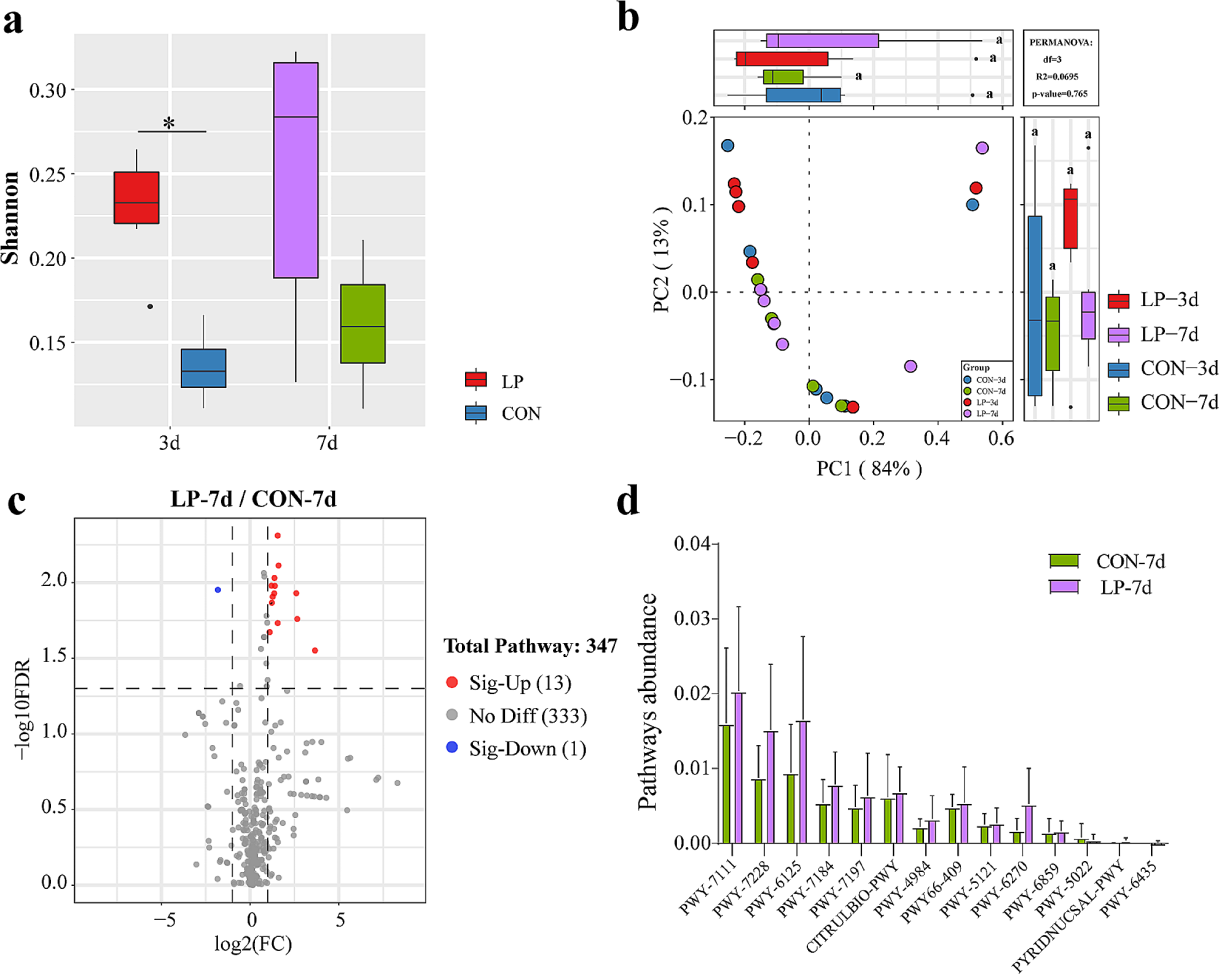
Analysis of metabolic pathway diversity in fecal samples of neonatal piglets. (**a**) Comparative box diagram of Shannon index. (**b**) Principal component analysis was performed based on bray distance. PCoA score plots are based on principal components 1 and 2 (at the metabolic pathway level). (**c**) Screening of differentially-expressed metabolic pathways between the two groups of 7-day-old piglets. Volcano plot showing the differences in metabolic pathway expression levels between the probiotic group (LP) and control group (CON). Red and blue dots indicate differentially expressed metabolic pathways significantly upregulated in the probiotic group (LP) and downregulated in the control (CON) group, respectively. (d) Detailed differentially expressed metabolic pathways in the fecal samples of 7-day-old neonatal piglets in the two groups

To further proceed diversity analysis of metabolic pathways to explore the effect of spraying *L. plantarum* P-8 fermentation broth on the metabolic pathways (Fig. 4a-b). Compared with the control group, alpha diversity analysis (Shannon index) revealed a higher metabolic pathway activity in the probiotic group among the 3-day-old piglets, however, the difference disappeared in 7-day-old piglets. Beta diversity (shown by PCoA and PERMANOVA) revealed no clear trend of separation between the gut microbiota metabolic pathway of the probiotic and control groups. These showed that spraying *L. plantarum* P-8 fermentation broth increased the diversity but did not affect the structure of metabolic pathways.

A total of 14 metabolic pathways showed significant differences between the two groups in 7-day-old piglets. Of these, 13 metabolic pathways (PWY-7717, PWY-7228, PWY-6125, PWY-7184, PWY-7197, PWY-4984, PWY-409, PWY-5121, PWY-6270, PWY-6859, PWY-6435, CITRULBIO-PWY, PYRIDNUCSAL-PWY) showed a positive fold difference (LP/CON) and are mainly related to the urea cycle, L-citrulline synthesis, and protein modification. One metabolic pathway, PWY-5022, showed a negative fold difference (LP/CON) and is associated with aminobutyric acid metabolism (Fig. 4c-d).

### Gut microbiota participated in the regulation of urea cycle and protein modification

**Fig. 5:**
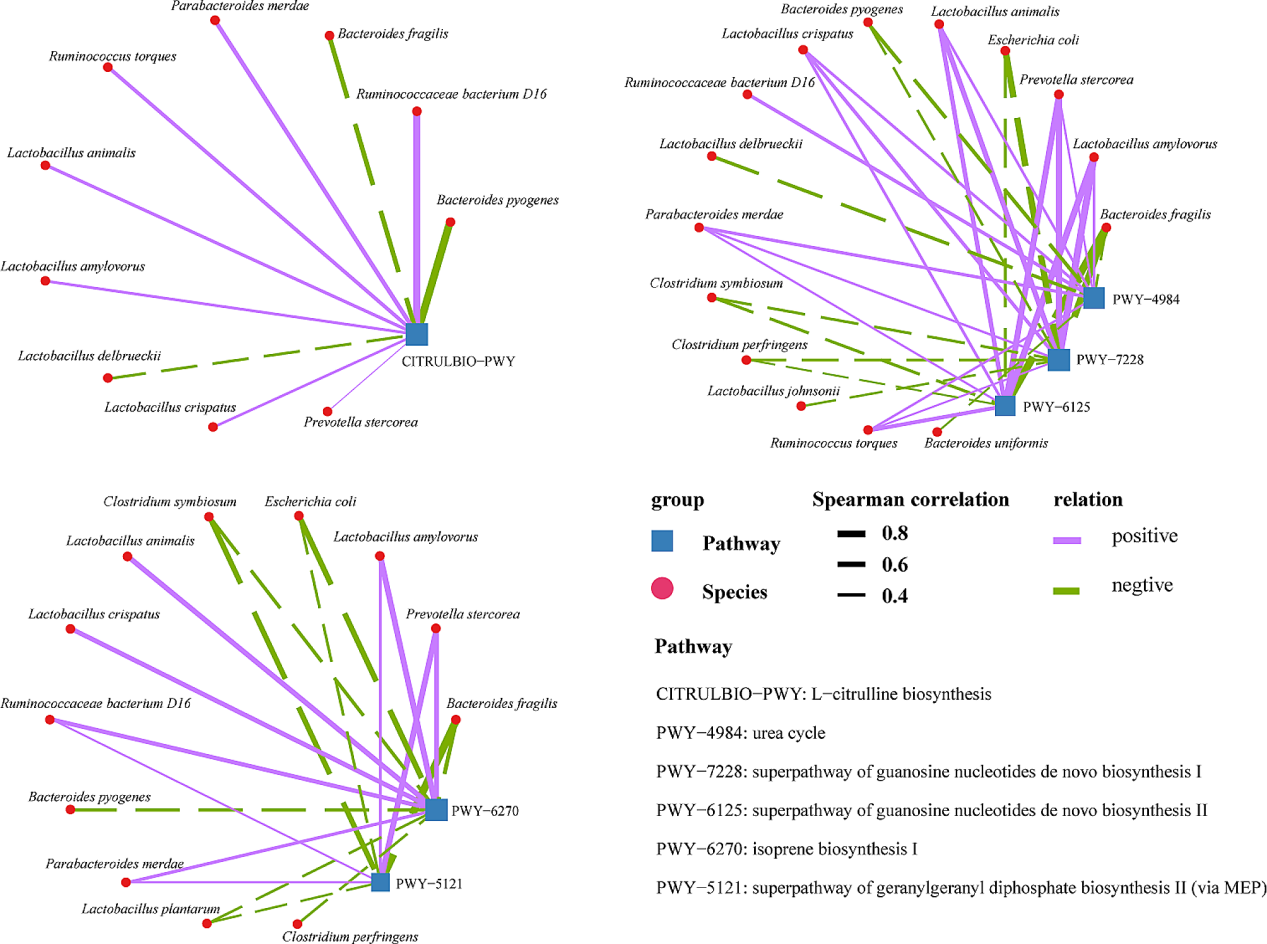
Correlation analysis between gut microbiota and metabolic pathways. Correlation network diagrams of (**a**) bacterial species and L-citrulline synthesis, (**b**) bacterial species and the urea cycle, and (**c**) species and protein modification. Purple and green lines represent the positive and negative correlations, respectively. Thick and thin solid lines represent correlations with absolute values > 0.8 (|r| > 0.8) and < 0.4 (|r| < 0.4), respectively

Interconnection networks between species-level and metabolic pathways was constructed (Fig. 5). It was found that 10 species were related to the synthesis of L-citrulline (CITRULBIO-PWY); of these, 7 species showed positive and 3 showed negative correlations, respectively. 15 species were associated with 3 metabolic pathways (PWY-4984, PWY-7228, PWY-6125) of the urea cycle; of these, 7 species showed positive and 8 showed negative correlations, respectively. 12 species were associated with protein modification (PWY-6270, PWY-5121), including 6 positive and 6 negative correlations.

### RFM construction evaluated the contribution of species

**Fig. 6:**
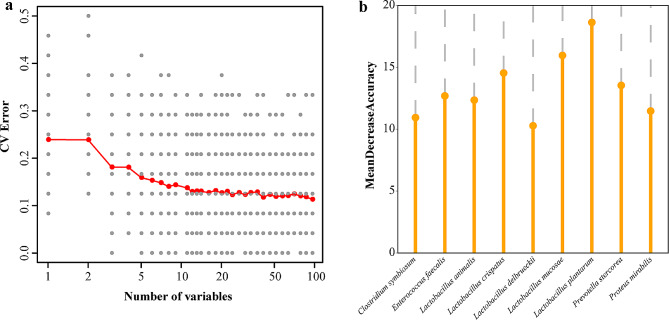
Construction of stochastic models and detailed species contribution

To explore the changes in gut microbiota and the importance of specific bacterial species during the growth of neonatal piglets, RFM was used to calculate the contribution of bacterial species in the two groups (Fig. 6). These species were then sorted based on high to low contribution to select the top strains and calculate CV Error. In total, 81 species were selected, and the accuracy of the prediction model was the highest. The 9 species, *L. plantarum, L. mucosae, Lactobacillus crispatus, Prevotella stercorea, Enterococcus faecalis, Lactobacillus animalis, Proteus mirabilis, Clostridium symbiosum*, and *L. delbrueckii*, made the highest contribution.

## Discussion

With the continuous development of science and technology, people pay more attention to the healthy breeding of animal husbandry. The neonatal period is a critical time for the establishment of gut microbiota in piglets, which plays a crucial role in animal growth and development [9]. Adding probiotics to feed could effectively maintain the balance of gut microbiota, improve the immune ability of animals and promote the healthy development of animal husbandry. This study showed that spraying *L. plantarum* P-8 fermentation broth effectively promoted the growth of neonatal piglets, and regulated the antioxidant capacity and immunity in neonatal piglets. *L. plantarum* P-8 adjusted the gut microbiota structure and increased beneficial bacteria, and maintained the gut microecological balance of neonatal piglets.

Our results found that spraying *L. plantarum* P-8 fermentation broth improved the growth of neonatal piglets and reduced the mortality to a certain extent. This phenomenon could be attributed to the successful establishment of initial gut microbiota. A well-established gut microbiota accelerates digestion and absorption of nutrients enabling faster growth of neonatal piglets and reducing mortality. Increasing the weight of neonatal piglets can effectively shorten the slaughter time, improve the feed-to-meat ratio, and enhance their resistance to diseases. In addition, *L. plantarum* P-8 fermentation broth treatment increased the activities of CAT and SOD but reduced the levels of IL-2 and IL-6, and effectually regulated the antioxidant capacity and immunity of neonatal piglets. CAT and SOD protect against excessive levels of intracellular reactive oxygen species preventing cell and tissue damage [26]. Ma indicated that dietary supplementation of *L. plantarum* during pregnancy and lactation periods decreased plasma levels of IL-1β, IL-2, IL-6 and IFN-α in piglets [22]. Wang showed that *Lactobacillus* can enhance the immune system of piglets by inhibiting the expression of pro-inflammatory factors such as IL-6, IL-8 and IFN-α [27]. The same results were found in our study. Thus, supplementation of *L. plantarum* P-8 could be used as an effective way to regulate the antioxidant capacity and immunity of neonatal piglets, in order to promote the healthy development of neonatal piglets.

The early gut microbiota of piglets is affected by many environmental factors, including the changes in parturition mode [28], diet [29], and feeding environment [30]. Spraying the fermentation broth changed the growth environment of neonatal piglets adjusting the overall structure of gut microflora and improving gut homeostasis. Chen indicated Firmicutes and Bacteroidetes were found to be the two main phyla in the intestine of neonatal piglets [31]. Bian showed *Fusobacterium*, *Clostridium*, and *Lactobacillus* were found to be the most enriched genera in the gut microbiota of neonatal piglets [32]. The same results were obtained in our study. We found that *F. mortiferum*, *C. symbiosum*, *Lactobacillus johnsonii*, *Lactobacillus amylovorus* and *L. delbrueckii* were enriched in the gut of neonatal piglets. Notably, *Lactobacillus* is one of the main and highly safe probiotics in functional foods [33]. It not only competes with pathogens but also improves the antioxidant and immunity status of host piglets [34]. Yang showed that supplementation of *L. plantarum* and *Pediococcus acidilactici* in diets can improve ADG, and significantly increase short-chain fatty acid-producing bacteria such as *Megasphaera*, *Roseburia* and *Faecalibacterium* in the gut of nursery pigs [35]. Yu indicated *L. plantarum* P-8 significantly increased the abundance of *L. plantarum*, and improved antioxidant capacity and growth performance in weaned piglets [19]. Liu showed that orally administrated *Lactobacillus casei* and *Enterococcus faecalis* could adjust gut microbiota and improve microbial similarity coefficients for keeping suckling piglet gut microbiota stable [36]. Our research had found *L. plantarum* P-8 significantly increased the relative abundance of *L. delbrueckii* and *L. mucosae*. Studies have shown that *L. delbrueckii* has good probiotic properties. It not only promotes anti-inflammatory effects but also improves metabolism and secretion of extracellular polysaccharides that can prevent/treat human and animal inflammatory bowel diseases [37]. *L. mucosae* can transiently adhere to the gut wall due to its mucus-binding gene (MUB) and colonize the host mucus layer or gut epithelial cells, in order to exert probiotic effects [38]. Our results were consistent with the results of most studies, which also found that *L. plantarum* could significantly increase the beneficial bacteria in the gut of piglets and improve the gut homeostasis of piglets. The difference was that *L. plantarum* P-8 could significantly increase *L. delbrueckii* and *L. mucosae*, which proves that there may be synergistic effects between *L. plantarum* P-8 and *L. delbrueckii* and *L. mucosae*. Our findings provide a new thinking to explore the mechanism of Lactobacillus plantarum exerting probiotic effect in the gut of neonatal piglets. The synergistic effect between *L. plantarum* P-8 and gut beneficial bacteria may be the key to improve the gut health and homeostasis of neonatal piglets.

Among the 3-day-old piglets, the diversity of gut microbiota metabolic pathways was significantly higher in the probiotics group than in the control group. This suggests a better gut microbiota function in the probiotic group. Studies have shown that *Lactobacillus* and *Bacteroides* are mainly responsible for degrading fructans and lactose in the gut of piglets. The exclusive presence of extracellular fructose (FruA) in *Lactobacillus* species represented the special nutrient needs of piglets during growth [39]. The correlation analysis revealed that *L. animalis*, *L. amylovorus*, and *L. crispatus* positively correlated with the L-citrulline synthesis pathway, while *L. delbrueckii*, *Bacteroides fragilis*, and *Bacteroides pyogenes* were negatively correlated with the L-citrulline synthesis pathway. This suggests that gut microbes *Lactobacillus* and *Bacteroides* and L-citrulline synthesis are closely related in neonatal piglets. L-citrulline plays a vital role in the pulmonary circulatory system of neonatal piglets. Studies have shown that oral L-citrulline can relieve nitric oxide synthesis by recombining with nitric oxide synthase relieving pulmonary hypertension in neonatal piglets [40]. Another study found that L-citrulline can regulate T cells (Treg) by upregulating the levels of IL-10 improving the immune system in mice [41]. Spraying *L. plantarum* P-8 fermentation broth improved the synthesis of L-citrulline promoting the physiological status of neonatal piglets. Also, we found that *L. crispatus*, *L. animalis*, and *L. amylovorus* of the Lactobacillus genus positively correlated with the urea cycle, while *B. pyogenes* and *B. fragilis* belonging to *Bacteroides* showed a negative correlation. The urea cycle is vital for nitrogen metabolism to remove the toxic effect of ammonia [42]. Spraying *L. plantarum* P-8 fermentation broth improved the urea cycle by changing the gut flora of neonatal piglets. Likewise, *L. animalis*, *L. amylovorus*, and *L. crispatus* showed a positive correlation with the protein modification process, while *B. fragilis* was negatively correlated. Protein modification is important for regulating their functions [43]. Lack of food (or starvation) affected the protein modification process in the gut microbiota of ABA (activity-based anorexia) mice, which was mainly caused by the order Clostridiales favoring ATP production [44]. Overall, it seems that spraying *L. plantarum* P-8 fermentation broth indirectly influenced with L-citrulline synthesis, urea cycle, and protein modification processes by regulating the gut microbiota of neonatal piglets, thereby affecting their physiological status. Notably, the RFM results suggested that *L. plantarum* and *L. mucosaus* were the most contributing bacteria to the gut microbiota of neonatal piglets. Again, it showed that *Lactobacillus* plays a crucial role in the establishment and maturation of gut microbiota in neonatal piglets.

It is necessary to acknowledge that this study had two important potential limitations. We have proposed for the first time the new intervention of spraying probiotics fermentation broth. In order to ensure the accuracy and reliability of the experimental results, it is necessary to repeat the experiment in the future to prove the effectiveness of spraying probiotic fermentation broth once again. Neonatal piglets were lactating. Since the feeding volume of neonatal piglets could not be measured, the conversion rate of breast milk intake to body weight of neonatal piglets was not evaluated in this experiment. In future, we will focus on this aspect. The effect of probiotics on the growth performance of neonatal piglets was evaluated by measuring the conversion rate of breast milk intake to body weight of neonatal piglets. Overall, compared with other intervention methods such as adding probiotics to the diet or gavaging, spraying probiotics fermentation broth as a new probiotic intervention method by improving the environmental medium, not only solves the problem that the neonatal piglets are not adapted to the diet and insufficient intake of probiotics, but also eliminates the stress response or rejection to the body of neonatal piglets.

## Conclusions

Spraying *L. plantarum* P-8 fermentation broth in the farrowing room significantly improved the growth performance, regulated the antioxidant capacity and immunity and improved gut homeostasis of neonatal piglets. This treatment indirectly affected the synthesis of L-citrulline, the excretion of toxins and protein modification processes of neonatal piglets. Compared with traditional methods, spraying probiotic fermentation broth is an effective probiotic intervention to promote healthy growth of neonatal piglets.

### Electronic supplementary material

Below is the link to the electronic supplementary material.

Supplementary Material 1

## Data Availability

The corresponding sequence data set has been deposited in the National Center for Biotechnology Information (NCBI) Sequence Read Archive (SRA) database (Accession number: PRJNA801914; Link: https://www.ncbi.nlm.nih.gov/bioproject/PRJNA801914).

## Abbreviations

LP: probiotic group
CON: control group
PBS: Phosphate buffer saline
ADG: Average daily gain
T-AOC: Total antioxidant capacity
CAT: Catalase
SOD: Superoxide dismutase
GSH-Px: Glutathione peroxidase
GSH: Glutathione
MDA: Malondialdehyde
IgA: Immunoglobulin A
IgG: Immunoglobulin G
IgM: Immunoglobulin M
IFN-γ: Interferon-γ
TNF-α: Tumor necrosis factor-α
IL-1β: Interleukin-1β
IL-6: Interleukin-6
IL-2: Interleukin-2
IL-4: Interleukin-4
IL-10: Interleukin-10
PCoA: Principal co-ordinates analysis
LEfSe: LDA Effect Size
RFM: Random forest model
NCBI: National Center for Biotechnology Information
SRA: Sequence Read Archive
MUB: Mucus-binding gene
